# Adaptation and spectral enhancement at auditory temporal perceptual boundaries - Measurements via temporal precision of auditory brainstem responses

**DOI:** 10.1371/journal.pone.0208935

**Published:** 2018-12-20

**Authors:** Diana B. Geissler, Elke Weiler, Günter Ehret

**Affiliations:** Institute of Neurobiology, University of Ulm, Ulm, Germany; Universidad de Salamanca, SPAIN

## Abstract

In human and animal auditory perception the perceived quality of sound streams changes depending on the duration of inter-sound intervals (ISIs). Here, we studied whether adaptation and the precision of temporal coding in the auditory periphery reproduce general perceptual boundaries in the time domain near 20, 100, and 400 ms ISIs, the physiological origin of which are unknown. In four experiments, we recorded auditory brainstem responses with five wave peaks (P1 –P5) in response to acoustic models of communication calls of house mice, who perceived these calls with the mentioned boundaries. The newly introduced measure of average standard deviations of wave latencies of individual animals indicate the waves’ temporal precision (latency jitter) mostly in the range of 30–100 μs, very similar to latency jitter of single neurons. Adaptation effects of response latencies and latency jitter were measured for ISIs of 10–1000 ms. Adaptation decreased with increasing ISI duration following exponential or linear (on a logarithmic scale) functions in the range of up to about 200 ms ISIs. Adaptation effects were specific for each processing level in the auditory system. The perceptual boundaries near 20–30 and 100 ms ISIs were reflected in significant adaptation of latencies together with increases of latency jitter at P2-P5 for ISIs < ~30 ms and at P5 for ISIs < ~100 ms, respectively. Adaptation effects occurred when frequencies in a sound stream were within the same critical band. Ongoing low-frequency components/formants in a sound enhanced (decrease of latencies) coding of high-frequency components/formants when the frequencies concerned different critical bands. The results are discussed in the context of coding multi-harmonic sounds and stop-consonants-vowel pairs in the auditory brainstem. Furthermore, latency data at P1 (cochlea level) offer a reasonable value for the base-to-apex cochlear travel time in the mouse (0.342 ms) that has not been determined experimentally.

## Introduction

Animal vocalizations and human speech are often emitted in series of sounds. Basic perceptual distinctions in the time domain are made at boundaries near 20–30 ms, 100 ms, and 400 ms inter-sound intervals (ISIs) and/or sound durations. Examples for boundaries near 20–30 ms and 100 ms are given by the transitions from hearing the pitch of a sound series (ISIs shorter than about 20 ms) to hearing rough sounds (ISIs between about 20–100 ms) to hearing a sound rhythm (ISIs longer than about 100 ms) [1–4]. The 20–30 ms boundary has also been found in the discrimination of stop consonants via voice-onset-time [5–7], for the perception of temporal order [8], gaps in sounds [9,10], categorization of mouse pup ultrasounds by their mothers [11], and for spectral integration of frequency components starting and ending together within 20–30 ms to be perceived as a single stream of auditory objects by humans [12,13] and mice [14]. The 100 ms boundary has also been found in the perception of acoustic streams in humans [15] and in wriggling call perception in mice [16]. Reports about the 400 ms boundary are found in loudness summation and forward masking in humans [17–19] and wriggling call perception in mice [16]. Neural correlates of these perceptual boundaries in the time domain are unknown because they have not systematically been studied or not been studied at all [2,20,21]. Interestingly, however, hearing the differences in the sound quality at these perceptual boundaries does not require explicit learning and seems to be a rather general ability of mammals. Therefore, our hypothesis is that these boundaries may be based on features of sound processing in the auditory periphery up to the midbrain inferior colliculus (IC). In order to test this hypothesis in the mouse, we used the same sounds as stimuli for the auditory system as in the tests for perception of communication calls. As method of testing, we used the auditory brainstem response (ABR) which allows to record information from the whole auditory periphery in one approach.

**Fig 1:**
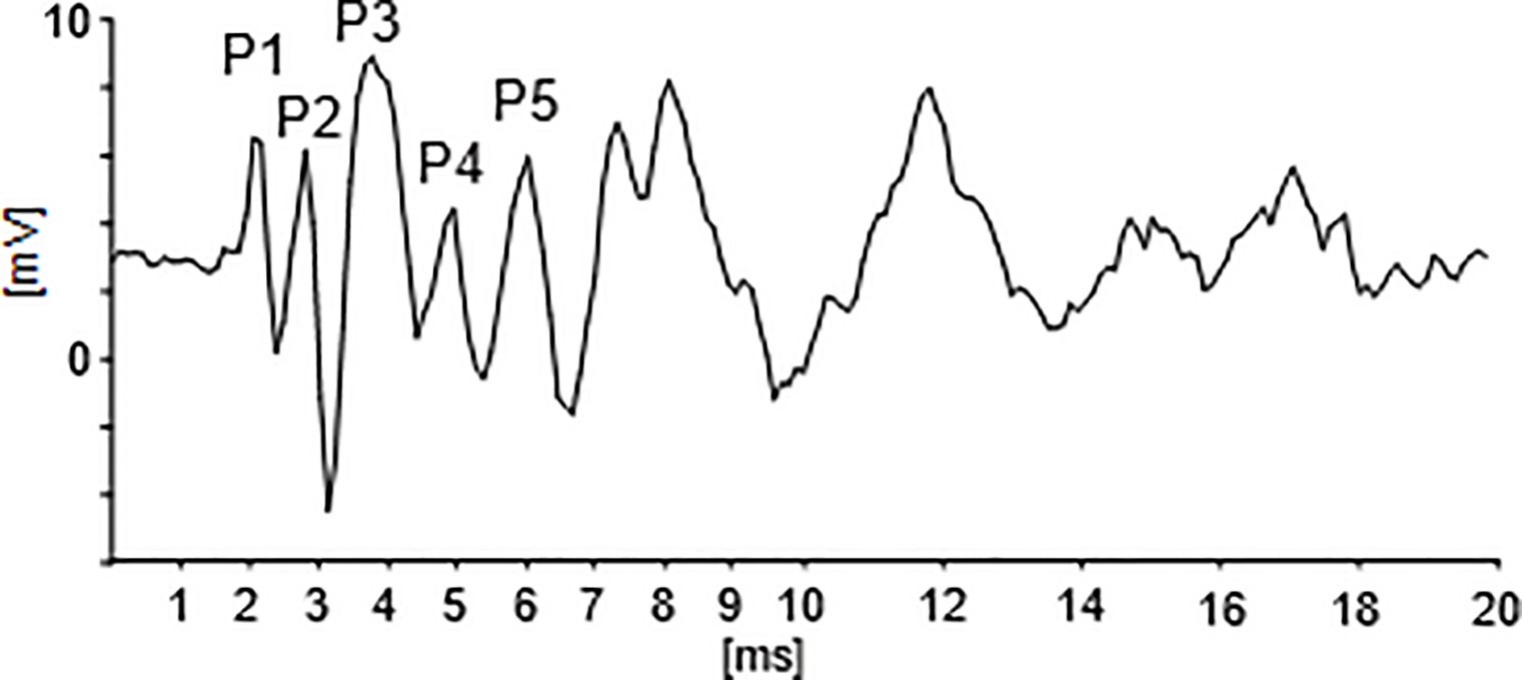
Example of an evoked-potential recording to 50 kHz tones. Only the first 20 ms after tone onset are shown. Five peaks (positive deflections, P1-P5) in the latency range of 1–6 ms define the mouse auditory brainstem response (ABR).

ABRs have widely been used as research tool to study auditory brainstem functions in humans and animals, including mice [22–31]. Auditory-evoked potential recordings in anesthetized mice usually consist of several waves (Fig 1) of which the first five peaks refer to the ABR. Each wave is thought to represent the sum of highly synchronous sound onset responses from cell populations in auditory brainstem centers. In the mouse, peak 1 (P1) seems to emanate from the cochlea (CO), i.e. from cochlear hair cells and the auditory nerve, peaks 2, 3, 4, 5 (P2, P3, P4, P5) mainly from cell groups in the cochlear nucleus (CN) ipsilateral to the stimulated ear, in the contralateral superior olivary complex (SOC), in the contralateral lateral lemniscus (LL) and in the IC, respectively [28,32–34]. Major topics of ABR studies in mice included development, aging, genetics, and pathologies of hearing simple sounds such as clicks, noise and tone bursts [28,31,35–45]. With the exception of speech in tests with humans [46–48], we are not aware of ABR studies using perceptually relevant communication sounds of animals or humans as stimuli. Since our primary focus was on perceptual boundaries in the time domain, we analyzed latencies and the precision of timing of ABR waves at the μs-level introducing a new measuring parameter for latency jitter. Main results concern the development of precision of temporal coding from the auditory periphery to the midbrain, correlates of the perceptual boundaries near 20–30 ms and 100 ms in adaptation at earlier and later ABR waves, respectively, and the enhancement of processing higher harmonics by low-frequency harmonics of sounds.

## Materials and methods

### Animals

Virgin female house mice (*Mus musculus*, outbred strain NMRI, 8–12 weeks old, 25 animals) from the breeding facilities of the Institute were used. After weaning, the animals were housed in female sibling groups. Food and water were available ad libitum. The experiments were carried out in accordance with the European Communities Council Directive (2010/63EU) and were approved by the appropriate authority (Regierungspräsidium Tübingen, Germany; number 1050).

### Experimental design and acoustic stimulation

**Fig 2:**
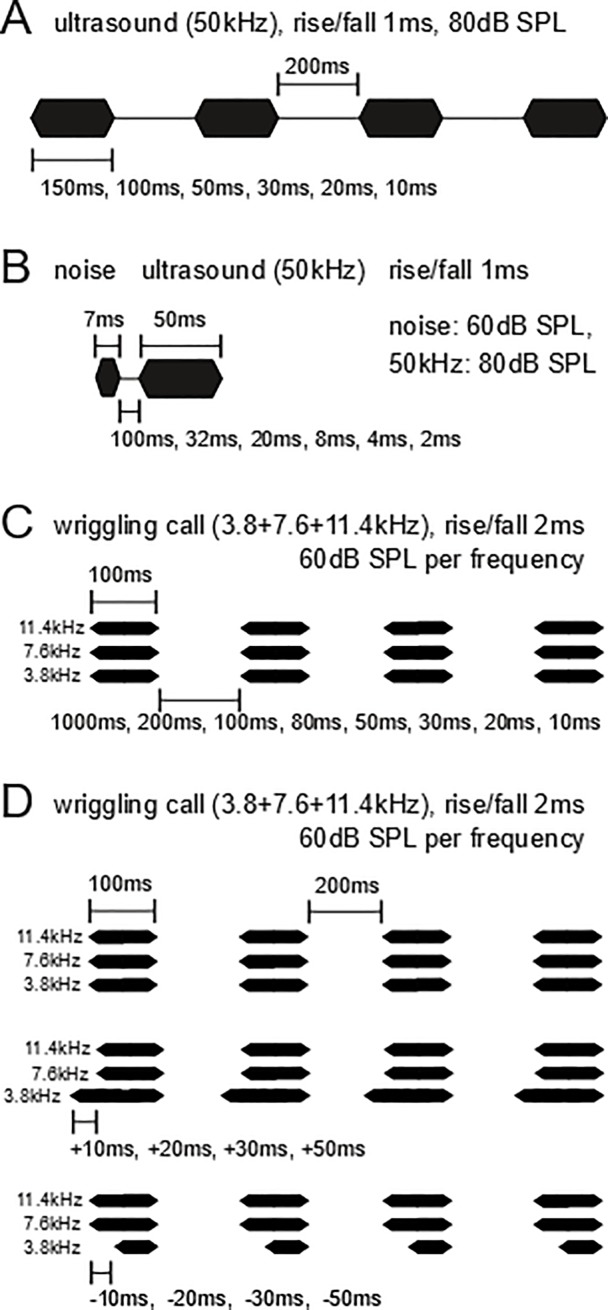
**Schemes of the acoustic properties of the sounds used in the four experiments (A-D).** In all experiments, the timing is varied as indicated: In (A), the tone duration in the series of four 50 kHz tones (models of mouse pup ultrasounds); in (B), the inter-stimulus interval (ISI) between the noise burst and the 50 kHz ultrasound; in (C), the ISI between the series of four models of mouse pup wriggling calls, each composed of three harmonics; in (D), series of four models of mouse pup wriggling calls with either simultaneous start of the three harmonics, or advanced or delayed start (with the indicated times) of the first harmonic (3.8 kHz).

Series of 50 kHz tones of 30 ms or longer duration and repetition rates 3-5/s effectively mimic mouse pup ultrasounds and release maternal behavior [11,49]. The 6 animals of experiment A were stimulated with 50 kHz tone bursts of the durations 150, 100, 50, 30, 20, 10 ms (1 ms rise and fall times included). Inter-stimulus intervals (ISIs) in series of four tone bursts were always 200 ms (Fig 2A).

When mouse pup ultrasounds are emitted, many of them are preceded by a short, soft (less than 70 dB) noise burst or click [50,51]. The 7 animals of experiment B were stimulated with 50 kHz tone bursts (50 ms duration, 1 ms rise and fall times included) preceded by bursts of white noise (7 ms duration, 1 ms rise and fall times included). The ISIs between the bursts of noise and ultrasound were 100, 32, 20, 8, 4 or 2 ms (Fig 2B). The stimuli consisting of noise burst + temporal gap + ultrasonic formant can be regarded as analogs of pairs of stop consonants and vowels of human speech.

Mouse pup wriggling calls consist of harmonically related main frequency components (formants) near 4, 8, and 12 kHz [52,53]. Behavioral tests with wriggling call models have shown the important features for the perception as relevant calls and for the release of maternal behavior [16,54]. These are three formants of 100 ms duration in series with ISIs of 100–400 ms duration [16]. The 7 animals of experiment C were stimulated with wriggling call models (100 ms duration, 2 ms rise and fall times included) consisting of 3.8, 7.6 and 11.4 kHz tones starting and ending simultaneously. In series of four call models, ISIs had durations of 1000, 200, 100, 80, 50, 30, 20 or 10 ms (Fig 2C).

In order to be perceived as relevant auditory objects for the release of maternal behavior, the three formants of wriggling call models have to start simultaneously within a time window of about 30 ms [14]. In group D (8 animals), the start of the fundamental frequency of 3.8 kHz was varied with regard to the onset of 7.6 + 11.4 kHz. Either 3.8 kHz started simultaneously with the two higher harmonics or earlier (+10, +20, +30, +50 ms) or later (-10, -20, -30, -50 ms). The three harmonics always ended simultaneously. The ISIs in a series of four call models had always durations of 200 ms relative to the 7.6 and 11.4 kHz harmonics (Fig 2D).

Tones and white noise were digitally generated with Signaltronic V 1.0 hard- and software (Albotronic, Oberkochen, Germany) in combination with a PC. Tones were initiated at zero phase. Ultrasounds (50 kHz tones) and the noise ran through an attenuator (RA 920A, Kenwood, Japan), filter (bandpass 1–100 kHz, 48 dB/octave; Kemo VBF8, Dartford, UK), 20 dB voltage amplifier (University of Ulm electronics) to an electrostatic speaker [55] with power supply (University of Konstanz electronics). The wriggling call models ran through the attenuator, filter, and a power amplifier (Denon PMA-1060, Nippon Columbia, Tokyo, Japan) to a dynamic speaker (Dynaudio D28, Dynaudio, Bensenville, USA). Sound pressure levels (re. 20 μPa) were measured (Bruel & Kjaer microphone 4135 with preamplifier 2633 plus measuring amplifier 2636; Bruel & Kjaer Instruments, Marlborough, MA) and adjusted to 80 dB for the ultrasound, 60 dB for the noise and 60.5 dB for each harmonic of the wriggling call models (70 dB total sound pressure level of the wriggling call models) at the right ear of the animal. On the basis of ABR threshold measurements in NMRI, C3H, and CBA mice [38,44,56] and very similar behavioral thresholds of NMRI and C3H mice [57], we assume that the above mentioned SPLs were about 10–20 dB above the ABR threshold of the NMRI mice at the frequencies used (3.8, 7.6, 11.4, 50 kHz). The perpendicular distance between loudspeakers and the ear entrance was 20 +/- 0.2 cm. This uncertainty of +/- 0.2 cm in the distance between ear and loudspeaker may have contributed +/- 6 μs to the measured latency variation between animals.

### ABR recording and data analysis

ABR recording took place in an anechoic and electrically shielded chamber. Animals were anesthetized by an intraperitoneal injection of an initial dose of 120 mg/kg ketamine (Ketavet, Bayer, Leverkusen, Germany) and 5 mg/kg xylazine (Rompun, Bayer, Leverkusen, Germany). Supplemental dosages of 25 mg/kg ketamine and 1 mg/kg xylazine were given as needed to maintain a motionless and pain-reflexless state of anesthesia. The body temperature of the mice was kept at 37°C by a feedback-controlled heating pad (Harvard, Holliston, MA, USA). Recording sessions lasted as long as the animals were in stable physiological conditions best visible in stable amplitudes of the ABR responses. Thus, the animals took part in one or two (3 animals) experiments run in one recording session. At the end of the recording session, each experimental animal, still anesthetized, died by another initial dose of anesthetic.

The ABR recordings were obtained with subdermal silver wire electrodes (diameter 0.25 mm) placed over the left-side IC (active), ventrolateral to the right ear (reference), and dorso-sacrum at the back of the mouse (ground). Electric potentials were amplified 10,000 times and 300 Hz– 3 kHz bandpass filtered (DAM 50; World Precision Instruments, Sarasota, USA), sent to a filter (Kemo VBF8, Dartford, UK) for another amplification (10 times) and filtering (1–3 kHz bandpass), fed in a CED 1401 Plus Interface (Cambridge Electronic Design, Cambridge, UK) controlled by a PC, and finally stored for averaging and analysis (Spike 2 software). Together with the ABR signals, trigger pulses marking the beginnings of the sound bursts and the sound signals themselves were also recorded in different channels via the CED 1401 Plus (sampling rate 125 kHz per channel) and stored in the computer. In addition, the ABR recordings and the sound signals and trigger pulses were monitored by an oscilloscope (type 2216, Tektronix, Wilmington, USA). Series of four sound bursts (experiments A, C, D) or pairs of sounds (experiment B) were repeated 500 times with 1 s pauses between the repetitions for averaging the responses.

From the ABR waveforms (Fig 1), we determined the peaks (P1, P2, P3, P4, P5) and measured their latencies with regard to sound onset. Peak latencies were calculated without the average runtime of 0.6 ms of the sound signals from the loudspeakers to the right ear of the animal. ABR waves of the shown quality (see related figures) were regularly obtained, so that the latencies of the wave peaks could usually be determined without doubt. In some recordings, two peaks became visible at the latency of an expected peak. In such cases, we took the peak with the higher amplitude as the relevant one. In cases, in which peak amplitudes were too small to be addressed or even absent, no latency value was taken. The accuracy with which latency measurement were obtained, was estimated by analyzing blind a second time 8 samples of ABR responses taken randomly from the recordings of experiments A-D. The latencies of the wave peaks P1-P5 were determined and compared with the results from the previous analyses of the same recordings. The 40 controls of latency measurements (8 samples x 5 peaks) led to differences of 0–16 μs (average 5 μs) compared with the previous data. Thus, we are confident that the data analysis did not produce errors larger than about +/- 8 μs. This variation equals the resolution of 16 μs to be expected by the 125 kHz sampling rate of the data.

### Statistical data analyses

Before statistical tests for possible differences between data sets were applied, the sets were checked for outliers [58,59] with α = 0.05. Identified outliers were not considered in the further statistical data evaluation. For comparing several groups of data with regard to possible differences in one parameter, one-way ANOVA or ANOVA on ranks (if data were not normally distributed and/or without equal variance) were applied. If the ANOVA indicated statistically significant differences, the final differences between the test groups as indicated in the text and figures were established by the paired t-test (concerning possible differences between responses to immediately following stimuli such as the four sound bursts in experiments A, C, D, or noise and ultrasound pairs in experiment B) or by the t-test or the U-test (Mann-Whitney rank sum test, when the data were not normally distributed). For other comparisons between two sets of data (means of latencies or SDs), t-tests or U-tests were used. If a data set was used in more than one statistical test, p-values were corrected according to Bonferroni [59]. SigmaPlot (11.0 software) was used for the statistical tests and regression analyses. All tests, including the significance of correlation coefficients, were two-tailed with significance level set at α = 0.05. Resulting p-values are indicated by stars in the figures as * p < 0.05, ** p < 0.01, *** p < 0.001.

## Results

### Experiment A: Exploration of the quality of ABR recording and analysis with acoustic models of mouse pup ultrasounds

**Fig 3:**
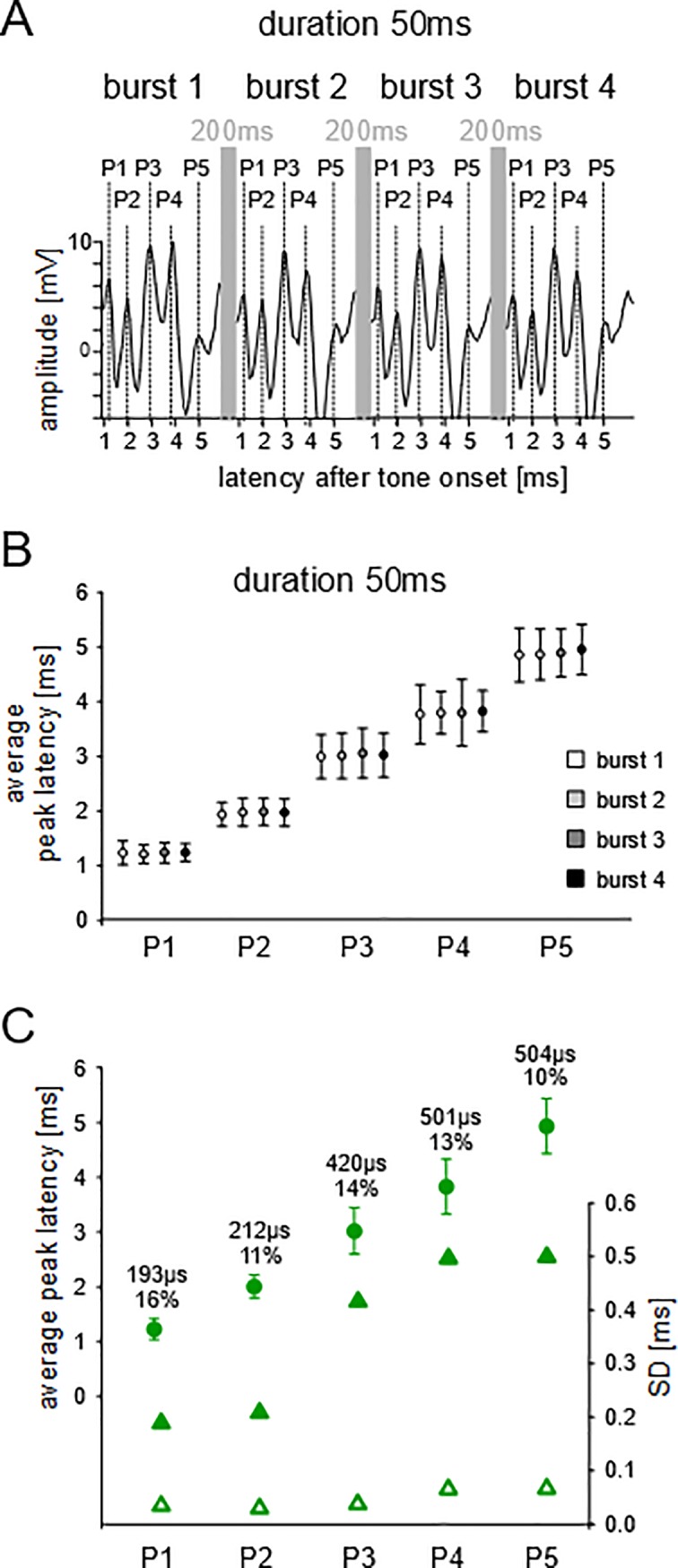
Experiment A, example ABR recording. **(A)** Each of the four 50 kHz tone bursts had a duration of 50 ms with 200 ms inter-stimulus-intervals (grey vertical bars). Wave peaks P1-P5 are indicated. (**B)** Average latencies with SDs to the four 50 kHz tone bursts (each of 50 ms duration) at the five wave peaks, P1-P5. (**C)** Grand average latencies (explanation, see text) with SDs to the stimuli of experiment A (filled circles) at the five peaks (P1-P5). The SDs of the means are also shown as values, relative values (%) and as filled triangles on the SD scale (right y-axis). The average SDs of the individual animals at the wave peaks are indicated by open triangles.

Fig 3A shows an ABR example to the series of 4 tone bursts of 50 ms duration each. All five peaks of the ABR waves (P1-P5) are clearly visible, and the ABR patterns to each tone burst are very similar. The ABR analyses of 6 animals resulted in the average latencies and the standard deviations (SDs) of the means for P1-P5 as shown in Fig 3B. Since neither the mean latencies to the 4 tone bursts of a given duration nor the mean latencies to the tone bursts of any of the 6 tested durations (Fig 2A) showed significant differences for any of the peaks (ANOVA; p > 0.2; see S1 Fig), latencies at a given peak were averaged across bursts and tone durations for each animal. These average values from the 6 animals were averaged to the grand average latency plotted in Fig 3C with the respective SDs for each peak (filled circles with SDs). Thus, the average peak latencies of Fig 3C (values are in Table 1) characterize population means together with their variation (SDs) among the animals. The mean latencies increased from P1 to P2 (0.777 ms), P2 to P3 (1.014 ms), P3 to P4 (0.811 ms), and P4 to P5 (1.104 ms) resulting in a total latency increase of 3.706 ms from P1 to P5 (see Table 1). The absolute SD values of the population means increased with increasing peak latencies (P1-P5) from about 200 to 500 μs (Fig 3C, filled triangles). The relative SDs of the peak latencies decreased from 16% (P1) to 11% (P2) and remained rather constant between 10% and 14% at the later peaks (Fig 3C).

**Table 1:** Summary of average wave latencies and latency differences between the peaks from the experiments A-D, and of statistical comparisons between data from the indicated experiments (C, D).

latencies [ms]	P1	P2	P3	P4	P5		P2-1	P3-2	P4-3	P5-4	P5-1
A 50kHz	1.233	2.009	3.023	3.834	4.938		0.777	1.014	0.811	1.104	3.706
D 3.8kHz	1.575	2.408	3.175	4.161	5.360		0.834	0.766	0.987	1.199	3.785
*Δ D—A*	*0*.*342*	*0*.*399*	*0*.*152*	*0*.*327*	*0*.*422*						
C, D no adaptation	1.544	2.394	3.207	4.151	5.304		0.850	0.813	0.944	1.153	3.760
C adaptation	1.549	2.561	3.422	4.491	5.563		1.012	0.861	1.069	1.072	4.014
D enhanced	1.422	2.285	3.140	4.005	5.306		0.863	0.855	0.865	1.301	3.884
C, D no adaptation vs. C adaptation	ns	p<0.02	p<0.01	p<0.001	ns	*Δ*	*0*.*162*	*0*.*048*	*0*.*125*	*-0*.*081*	*0*.*254*
C, D no adaptation vs. D enhanced	p<0.001	p<0.01	ns	p<0.01	ns						

Table 1 shows means of response latencies at the 5 wave peaks (P1-P5), differences of latencies between the peaks indicated (P2-1, P3-2, P4-3, P5-4, P5-1), differences between the values from experiment A 50 kHz and D 3.8 kHz (*Δ* D–A in italics), and differences between the peak difference values of ‘C, D no adaptation’ and ‘C adaptation’ (*Δ* in italics). The values of the row ‘C, D no adaptation’ are the means from experiment C to 1000, 200, 100 ms ISI, and from experiment D with synchronous start of the three harmonics and advanced start of the two higher harmonics. ‘C adaptation’ shows the values from the adapted responses in experiment C. ‘D enhanced’ shows the means from the cases of two higher harmonics with preceding 3.8 kHz. Results of statistical comparisons of mean latencies between the experiments are also shown with the respective p-values; ns = non significant (further explanation, see text).

The above mentioned averaging of latencies across the responses to the four tone bursts and all tone durations provided not only the individual means but also SDs for each individual animal. From these individual SDs, we calculated the average individual SDs at the wave peaks across the animals and plotted the average values also in Fig 3C (open triangles). The average individual SDs were very small i.e. 40 μs at P1, 35 μs at P2, 42 μs at P3, 71 μs at P4, and 72 μs at P5 (see Table 2). There were no significant differences of SDs between the peaks (ANOVA; p > 0.05).

**Table 2:** Summary of average standard deviations of the individuals from the experiments A-D, and of statistical comparisons between data from the indicated experiments (A, C, D).

SD individuals [ms]	P1	P2	P3	P4	P5					
A 50kHz	0.040	0.035	0.042	0.071	0.072	ns				
D 3.8kHz	0.067	0.042	0.052	0.056	0.070	ns				
C, D no adaptation	0.054	0.036	0.037	0.064	0.080	P4 > P1, P2, P3: p<0.05; P1 > P2: <0.05 P5 > P1, P2, P3: p<0.01				
C adaptation	0.090	0.048	0.043	0.109	0.079	P1, P4 > P2, P3: p<0.05				
D enhanced	0.043	0.030	0.037	0.064	0.082	P4, P5 > P1, P2, P3: p<0.01; P1 > P2: p<0.01				
C, D no adaptation vs. C adaptation	p<0.01	p<0.05	ns	p<0.01	ns					

Table 2 shows the means of standard deviations of latencies of the individuals (SD individuals) from the same experiments as mentioned in Table 1 for the mean latencies. Results of statistical comparisons of mean SDs of individuals between the peaks in experiments A and D and mean SDs of individuals between the experiments are also shown with the respective p-values; ns = non significant (further explanation, see text).

In summary, the average peak latencies were independent of sound duration and increased by about 1 ms with increasing ordinal peak number. Relative SDs of the means were largest at P1. The individual variation of onset responding expressed as average individual SDs was between 5–10 times smaller than the SDs of the mean peak latencies, reached values as small as 35 μs, and was rather independent of the level of processing from the CO to the IC.

### Experiment B: Mimicking VOT processing with pairs of noise bursts and ultrasounds

**Fig 4:**
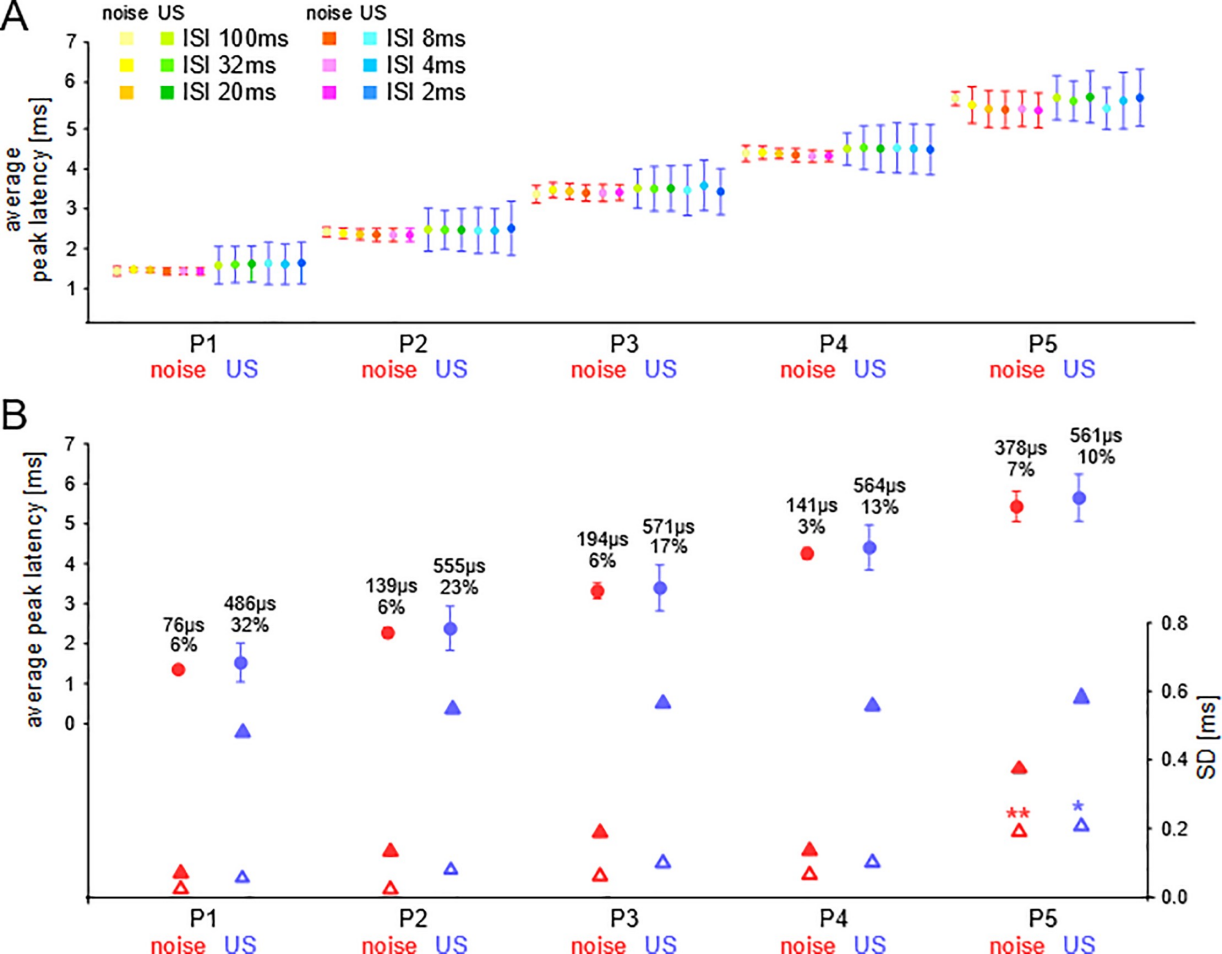
Experiment B, data evaluation. **(A)** Average latencies with SDs at the five wave peaks (P1-P5) in response to noise-ultrasound pairs color-coded for the tested ISIs. (**B)** Grand average latencies (explanation, see text) with SDs to the noise and ultrasound stimuli (filled circles) at the five peaks (P1-P5). The SDs of the means are also shown as values, relative values (%) and as filled triangles on the SD scale (right y-axis). The average SDs of the individual animals at the wave peaks are indicated by open triangles. The asterisks mark significantly larger values at P5 compared to those at P1 and P2.

Example ABR recordings to noise-ultrasound pairs with ISIs of 100, 20, 8, and 2 ms duration are shown in the S2 Fig. The results of the quantitative analyses of P1-P5 latencies of ABRs to noise and ultrasounds with ISIs of 2–100 ms as parameter are shown in Fig 4. In Fig 4A the peak latencies averaged from the ABRs of 7 animals are plotted together with the SDs of the means. For all noise-ultrasound pairs mean response latencies were independent of the ISIs at each wave peak (ANOVA; p > 0.2). In addition, mean latencies of noise responses did not differ from the means of ultrasound responses for any ISI and peak (paired t-test; p > 0.1). The SDs of the means of the noise responses were, however, significantly smaller than the SDs of the means of the ultrasound responses at all ISIs at all peaks (ANOVA on ranks followed by U-tests; p < 0.001 at P1-P4; p < 0.01 at P5; see Fig 4A).

Because ABR latencies to noise and ultrasounds were independent of the ISIs, latencies were averaged across all ISIs separately for each animal leading to individual means in response to noise and ultrasounds and individual SDs for each of the 5 peaks. The means of the 7 animals were then averaged separately for each peak and the grand average values with their SDs plotted in Fig 4B (filled circles with SDs). Mean latencies increased through the sequence of peaks from P1 to P5 resulting in a total latency increase of 4.074 ms and 4.105 ms for noise and ultrasound, respectively. There were no significant differences between the population means in response to noise and ultrasounds at any peak (U-test; p > 0.3). Absolute and relative values of the SDs of the population means are also given in Fig 4B. Absolute values increased from 76 μs at P1 to 378 μs at P5 (filled triangles with regard to noise) while relative SDs remained rather constant (about 6%). For ultrasound responses, absolute SD values did not increase systematically with the peak number (Fig 4B, filled triangles with regard to US) and varied between 486 and 571 μs. Relative SDs of the ultrasound means decreased from 32% at P1 to 10% at P5.

The average individual SDs of response latencies at P1-P5 are also shown in Fig 4B (open triangles). As in experiment A, average individual SDs were much smaller than the SDs of the group means (filled triangles). The absolute values were at P1 29 and 59 μs, at P2 24 and 83 μs, at P3 67 and 105 μs, at P4 61 and 107 μs, at P5 197 and 213 μs for noise and ultrasound responses, respectively. The values at P5 were significantly larger than the values at P1 and P2 for both noise and ultrasound responses (ANOVA on ranks followed by U-test; p < 0.01 noise, p < 0.05 ultrasound).

In summary, preceding noise at any ISI of 2–100 ms did not significantly change the ABR response latencies to the following 50 kHz ultrasounds at any peak (P1-P5). In addition, the grand average peak latencies in response to the ultrasounds in experiment B did not significantly differ from the grand average peak latencies in experiment A at any peak (t-test or U-test; p > 0.05). Preceding noise led, however, to general and significant increases of the variation of the mean latencies among the animals for all peaks (SDs of the means from experiment A vs. SDs of the means from experiment B ultrasound: t-test; p < 0.001 for P1, P2, P3; p < 0.05 for P4, P5). The average variation of latencies within the individual animals (SD individuals) was not generally increased in experiment B (ultrasound responses) compared to experiment A. Mean individual SDs in experiment B were significantly larger than those in experiment A at P2 (U-test; p < 0.05), P3 (t-test; p < 0.001), and P5 (U-test; p < 0.05).

### Experiment C: Testing perceptually relevant ISIs of mouse pup wriggling calls

**Fig 5:**
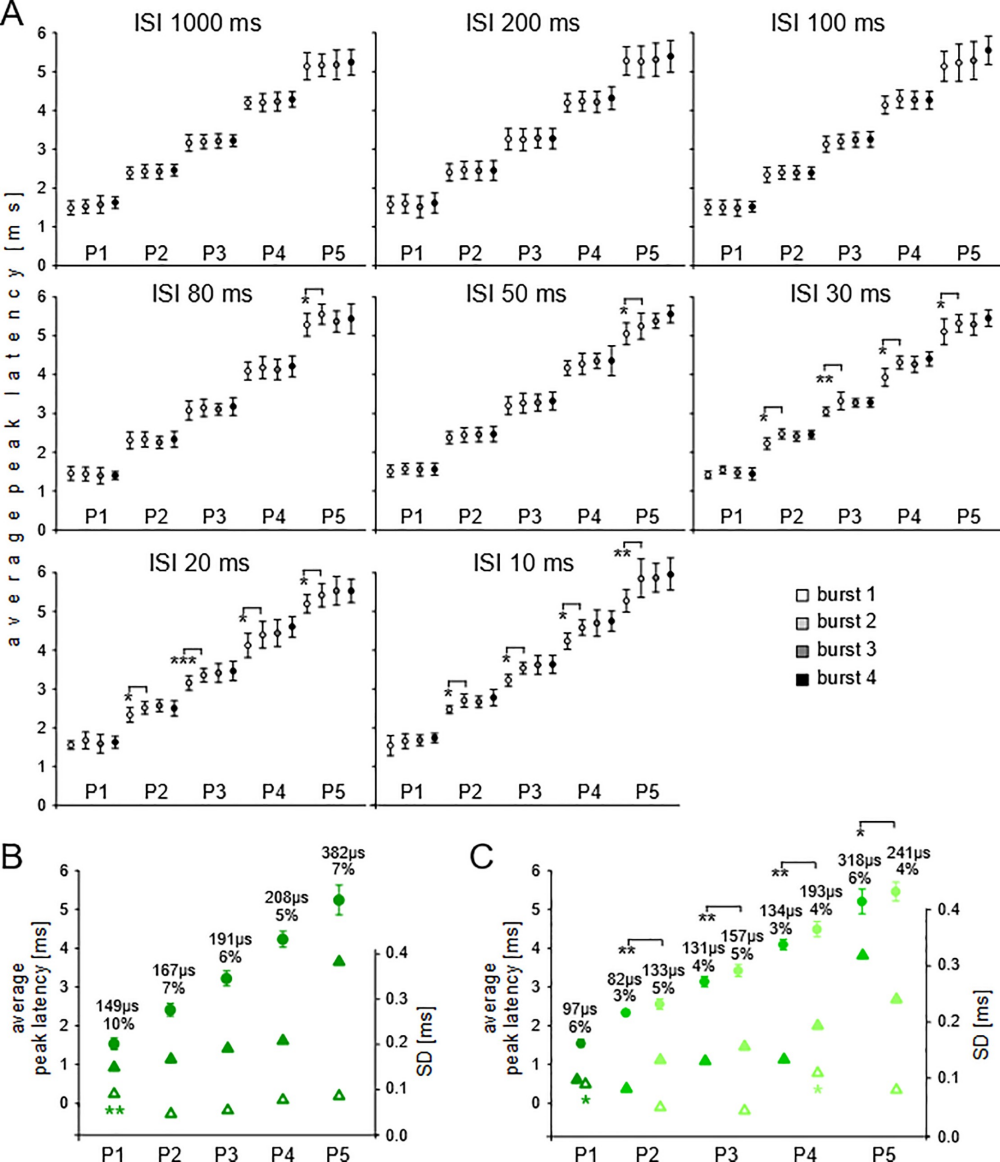
Experiment C, data evaluation. **(A)** Average latencies with SDs at the five wave peaks (P1-P5) in response to the series of four wriggling call models (bursts 1–4) with ISIs of 10–1000 ms between the sound bursts as indicated in each panel. Asterisks show significant differences of average latencies between bursts 1 and 2. (**B)** Grand average latencies (explanation, see text) with SDs to the wriggling call series with ISIs of 100, 200, 1000 ms (filled circles) at the five peaks (P1-P5). The SDs of the means are also shown as values, relative values (%) and as filled triangles on the SD scale (right y-axis). The average SDs of the individual animals at the wave peaks are indicated by open triangles. The asterisks mark significantly larger value at P1 compared to that at P2. (**C)** Grand average latencies (explanation, see text) with SDs to the wriggling call series with ISIs of 10–80 ms (filled circles) at the five peaks (P1-P5). Latencies of responses without significant latency increase (e.g. responses to all bursts at P1 and to burst 1 at other peaks; compare panel A and text) were averaged to the values shown in dark green, latencies of responses with significant latency increase were average to the values shown in light green. Asterisks indicate significant differences between dark and light green latency values. The SDs of the means are also shown as values, relative values (%) and as filled triangles on the SD scale (right y-axis). The average SDs of the individual animals at the wave peaks are indicated by open triangles. The asterisks mark significantly larger SD values at P1 and P4 compared to those at P2 and P3.

Example ABR recordings to series of four bursts of wriggling call models with ISIs of 200 and 20 ms duration are shown in the S3 Fig. The results of the quantitative analyses of response latencies are shown in Fig 5. Latency means with SDs of the means, averaged across 7 animals, are shown in Fig 5A for P1-P5 separately for the 4 sound bursts used as stimuli at the indicated ISIs (10–1000 ms). For ISIs of 1000, 200, and 100 ms, latency means to the 4 sounds bursts did not differ at any wave peak (ANOVA, p > 0.2). At ISIs of 80 and 50 ms, the means of the burst latencies at P5 were significantly longer in response to burst 2 compared to burst 1 (paired t-test; p < 0.05) as indicated in the figure. At 10, 20, and 30 ms ISIs, significant differences between the mean latencies of responses to bursts 1 and 2 occurred at all peaks (P2, P3, P4, P5; paired t-test, p < 0.01 or p < 0.05; see Fig 5A) except P1 (ANOVA, p > 0.2). In summary, except in the CO, short ISIs of 10, 20, and 30 ms led to significantly longer latencies to the second compared to the first sound in a series.

**Fig 6:**
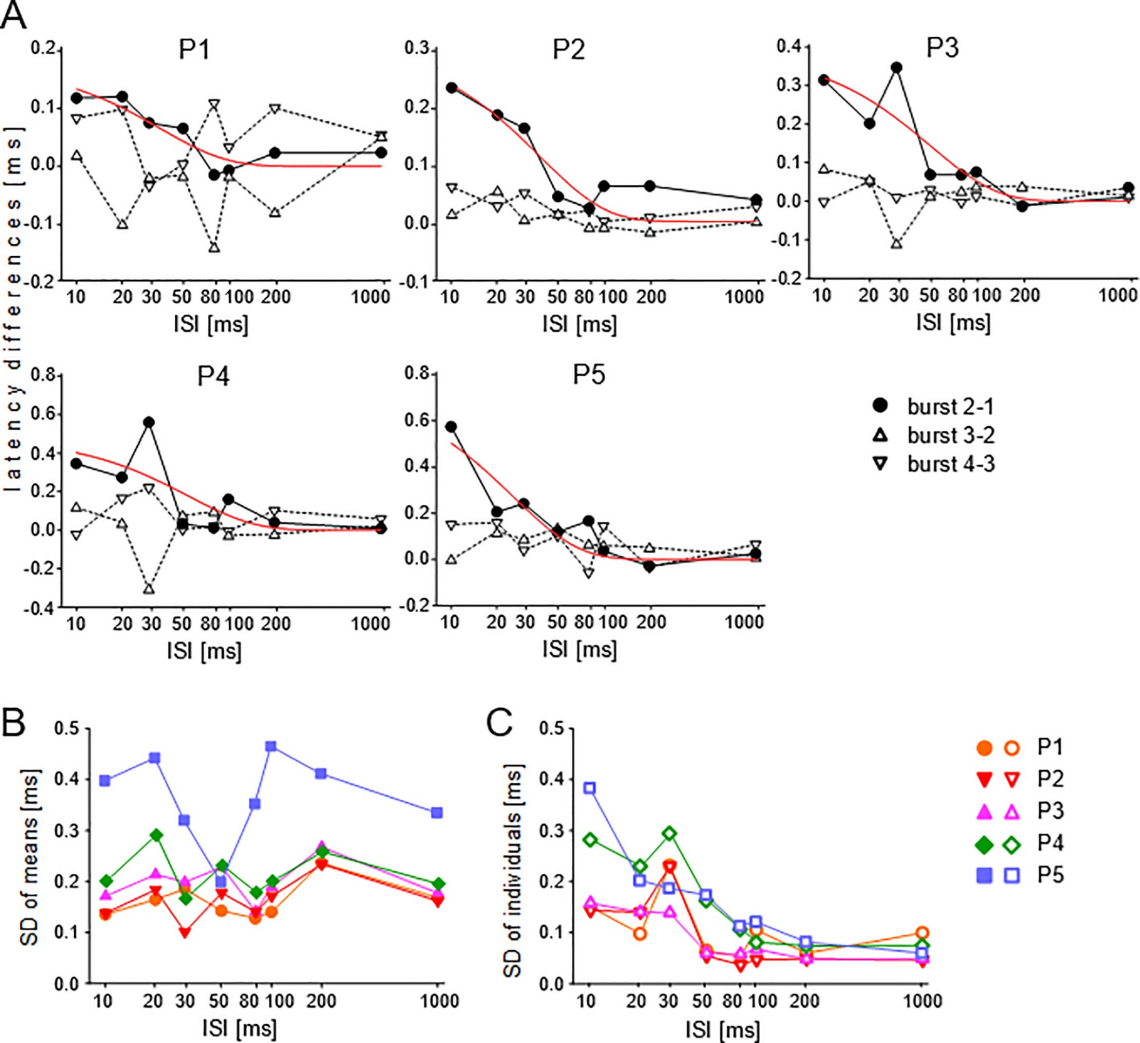
Experiment C, ISI dependence of latencies and latency variation. **(A)** Differences between average latencies to the sound bursts (burst 2–1, burst 3–2, burst 4–3) are plotted as function of the ISIs. The significant increases of the latency difference between bursts 1 and 2 with decreasing ISI is modelled by exponential decay functions (red curves) the parameters of which are given in Table 3. (**B)** SDs of averaged latencies to the four sound bursts are plotted as function of the ISIs with wave peaks as parameter. Systematic relations were not present. (**C)** Average individual SDs of response latencies to the four sound bursts are plotted as function of the ISIs with wave peaks as parameter. The significant decreases of the SDs with increasing ISIs are modelled by decay functions the parameters of which are given in Table 3.

Fig 5A shows that average response latencies to the four sound bursts may differ at certain ISIs and peaks. Whether latency differences in the responses to the sound bursts varied systematically with ISI duration is shown in Fig 6A. Latency differences of responses to bursts 1 and 2 (burst 2–1), bursts 2 and 3 (burst 3–2), and bursts 3 and 4 (burst 4–3) from the 7 animals were averaged and plotted as function of the ISIs, separately for each peak (P1-P5). At all five peaks, burst 2–1 latency differences had positive values for short ISIs which decreased, on average, towards zero for longer ISIs. The decrease of the burst 2–1 values with increasing ISIs was statistically significant (nonlinear regression, y = b e^-ax^) at all peaks with p-values between 0.0383 and 0.0017 (compare Table 3). At short ISIs, Fig 6A shows an increase of the burst 2–1 latency difference from P1 to P5, i.e. with 10 ms ISIs, the burst 2–1 latency difference was about 0.13 ms at P1, 0.23 ms at P2, 0.32 ms at P3, 0.40 ms at P4, and 0.50 ms at P5. In contrast, burst 3–2 and burst 4–3 latency differences were very variable at all peaks and without significant relationships to ISIs (Fig 6A). This result adds to the presentation of the data in Fig 5A showing significant latency increases with shortening of the ISIs which were specific at each level of processing from the auditory periphery to the midbrain and the larger the higher the level of processing was. These significant latency increases concerned, however, only the response to the second relative to the first sound burst in a series.

**Table 3:** Regression functions of the ISI dependences at the wave peaks P1-P5 shown for average differences of response latencies between bursts 2–1 in Fig 6A and for SDs of individuals in Fig 6C.

`	ISI dependence of latency differences, burst 2–1 [ms] (Fig 6A)			ISI dependence of SD individuals [ms] (Fig 6C)		
peak	ISI (x), difference (y), regression	r (DF)	p	ISI (x), SD (y), regression	r (DF)	p
P1	y = 0.1769 e ^-0.0283x^	0.9012 (6)	< 0.01	y = 0.0758 + 0.2709 e ^-0.1297x^ y = 0.246–0.105 log x	0.8162(6) 0.975 (2)	= 0.1114 < 0.05
P2	y = 0.3067 e ^-0.0264x^	0.8941 (6)	< 0.01	y = 0.0407 + 0.1676 e ^-0.0388x^ y = 0.289–0.133 log x	0.9626(6) 0.955 (2)	< 0.001 < 0.05
P3	y = 0.3915 e ^-0.0200x^	0.8766 (6)	< 0.01	y = 0.0503 + 0.1676 e ^-0.0394x^ y = 0.389–0.126 log x	0.9760 6) 0.966 (2)	< 0.001 < 0.05
P4	y = 0.4836 e ^-0.0184x^	0.7336 (6)	< 0.05	y = 0.0695 + 0.2689 e ^-0.0244x^ y = 0.485–0.198 log x	0.9953 6) 0.995 (3)	< 0.001 < 0.001
P5	y = 0.7465 e ^-0.0395x^	0.9092 (6)	< 0.01	y = 0.1047 + 0.6416 e ^-0.0857x^ y = 0.435–0.145 log x	0.9511(6) 0.869 (5)	< 0.001 < 0.05

All functions express the decrease of adaptation effect with increasing ISI duration. r, correlation coefficient; DF, degrees of freedom; p, level of statistical significance of r.

As in experiment A, latencies across the four bursts with ISIs without significant latency shifts at any peak (ISIs of 1000, 200, 100 ms; Fig 5A) were averaged for each animal. The means from the 7 animals were averaged to the grand average latency plotted in Fig 5B with the respective SDs of the means for each peak (filled circles with SDs). The means significantly increased through the sequential peaks from P1 to P5 resulting in a total latency increase of 3.705 ms. Absolute values of SDs increased with peak number from 149 μs at P1 to 382 μs at P5 (filled triangles, Fig 5B). The relative SD value of 10% at P1 decreased to 5–7% at the other peaks. Averaging latencies across the responses to the four sound bursts and ISIs of 1000, 200, and 100 ms provided also SDs for each individual animal. These individual SDs were averaged separately for each peak across the animals and the values plotted in Fig 5B (open triangles). Average individual SDs were again much smaller than the SDs of the means (open vs. closed triangles in Fig 5B). The average individual SD at P1 (90 μs) was significantly larger (p < 0.01) than that at P2 (46 μs) but did not differ from those at P3 (54 μs), P4 (77 μs), and P5 (86 μs) (ANOVA followed by t-tests).

Sound burst series with ISIs of 80 ms and shorter showed for some peaks significant latency increases in responses to the second compared to the first burst in a series (Fig 5A). These differences led to further analyses of ABRs in response to sound series with these ISIs. Since latencies at P1 did not significantly increase to the second, third and forth sound burst in the series (Fig 5A), P1 latencies were averaged across the four bursts and the responses to the ISIs of 10–80 ms for each animal and then these average values from the 7 animals were averaged to the grand average latency at P1 plotted with the SD of the mean value in Fig 5C. At P2, P3, and P4, latencies to the first bursts were averaged across the responses to the ISIs of 10, 20, and 30 ms for each animal and the grand average values for the 7 animals plotted with the SDs of the means in Fig 5C (filled circles with SDs in dark green related to P2, P3, P4). At P2, P3, and P4, latencies to the second, third, and forth bursts were also averaged for each animal across the bursts and the ISIs of 10, 20, and 30 ms and the grand average values for the 7 animals plotted with the SDs of the means in Fig 5C (filled circles with SDs in light green related to P2, P3, P4). The means are also given in Table 1 (‘C adaptation’). At P5, the same procedure as at P2-P4 was applied with the inclusion of the recordings at 50 and 80 ms ISIs (Fig 5C, filled circles with SDs in light green related to P5; value in Table 1, ‘C adaptation’ at P5). Fig 5C shows that mean latencies in response to the first sound burst in a series were significantly shorter than the latencies to the following bursts at P2-P5 (t-test; p < 0.01 or p < 0.05 as indicated). Absolute values of the SDs of the means increased from P2 (82 μs) to P5 (318 μs; filled triangles in dark green, Fig 5C). Absolute values of the SDs of the means from the responses to the second, third and forth bursts also increased from P2 (133 μs) to P5 (241 μs; filled triangles in light green, Fig 5C). Relative SDs of all means varied between 3% and 6%. The individual SDs of the animals related to the latency means at P1 and, at the other peaks, to the means obtained for the second, third and forth bursts in the sound series were used to calculate the average individual SDs. The values are also shown in Fig 5C (open green triangles) and in Table 2 (‘C adaptation’). The average individual SDs at P1 and P4 were significantly larger than those at P2 and P3 (ANOVA on ranks followed by U-Test; p < 0.05).

Fig 6A shows latency changes at the wave peaks P1-P5 as function of the duration of the ISIs between the four sound bursts. Whether changes also existed for the standard deviations of the latencies is shown in Fig 6B and 6C. In Fig 6B, the SDs of the mean latencies to the four sound bursts averaged across the responses of the seven animals are shown separately for all wave peaks as a function of the ISI duration. Although SDs of the means were quite variable, especially at P5, general trends (increases or decreases) with changing ISIs did not occur. The average individual SDs of the seven animals, however, decreased at each peak with increasing duration of the ISIs (Fig 6C). At P1 the average SDs at 10 ms and 1000 ms ISI differed by 0.041 ms, at P2 by 0.098 ms, at P3 by 0.109 ms, at P4 by 0.207 ms, and at P5 by 0.324 ms. The functions were modeled by the nonlinear regression y = y_0_ + b e^-ax^. Excluding the values at 30 ms ISI, which were at P1, P2, and P4 at odds with the general trend, the equations listed in Table 3 were obtained. Except at P1, the regressions were statistically significant with p < 0.05 to p < 0.001 (Table 3). The decreasing part of the exponential function can also be described by a linear regression of the form y = y_0_ –a log x. Excluding again the values at 30 ms ISI, the decreasing part of the exponential function comprised ISIs of 10–80 ms at P1, P2 and P3, of 10–100 ms at P4, and of 10–1000 ms at P5. All regressions with the equations listed in Table 3 were statistically significant with p < 0.05 or p < 0.001. Inserting the y_0_-values of the exponential decrease functions for each peak (Table 3) as y in the respective linear regression functions, the x-values could be obtained at which the exponential decrease reached the constant y_0_ level. The resulting ISI values were 42 ms for P1, 74 ms for P2, 78 ms for P3, 125 ms for P4, and 190 ms for P5.

In summary, the length of ISIs between identical sounds (wriggling call models) systematically influenced both the latency of sound onset responses and the precision of timing of the responses (latency jitter expressed by SDs of individuals) at all levels of the auditory pathway represented by the ABR waves in very similar ways. This newly found correspondence of response latency and latency variation in the context of adaptation (see Discussion) shows significant latency adaptation for ISIs shorter than about 30 ms in the auditory periphery, shorter than 50 ms in brainstem centers, and shorter than 100 ms at the suggested IC level. Adaptation of the latency jitter covered slightly longer ISIs than the latencies themselves, namely about 42–190 ms. The largest loss of precision was observed for the shortest tested ISI of 10 ms at P4 and P5, the smallest at P1.

### Experiment D: Testing properties of time-critical spectral integration in wriggling call perception

**Fig 7:**
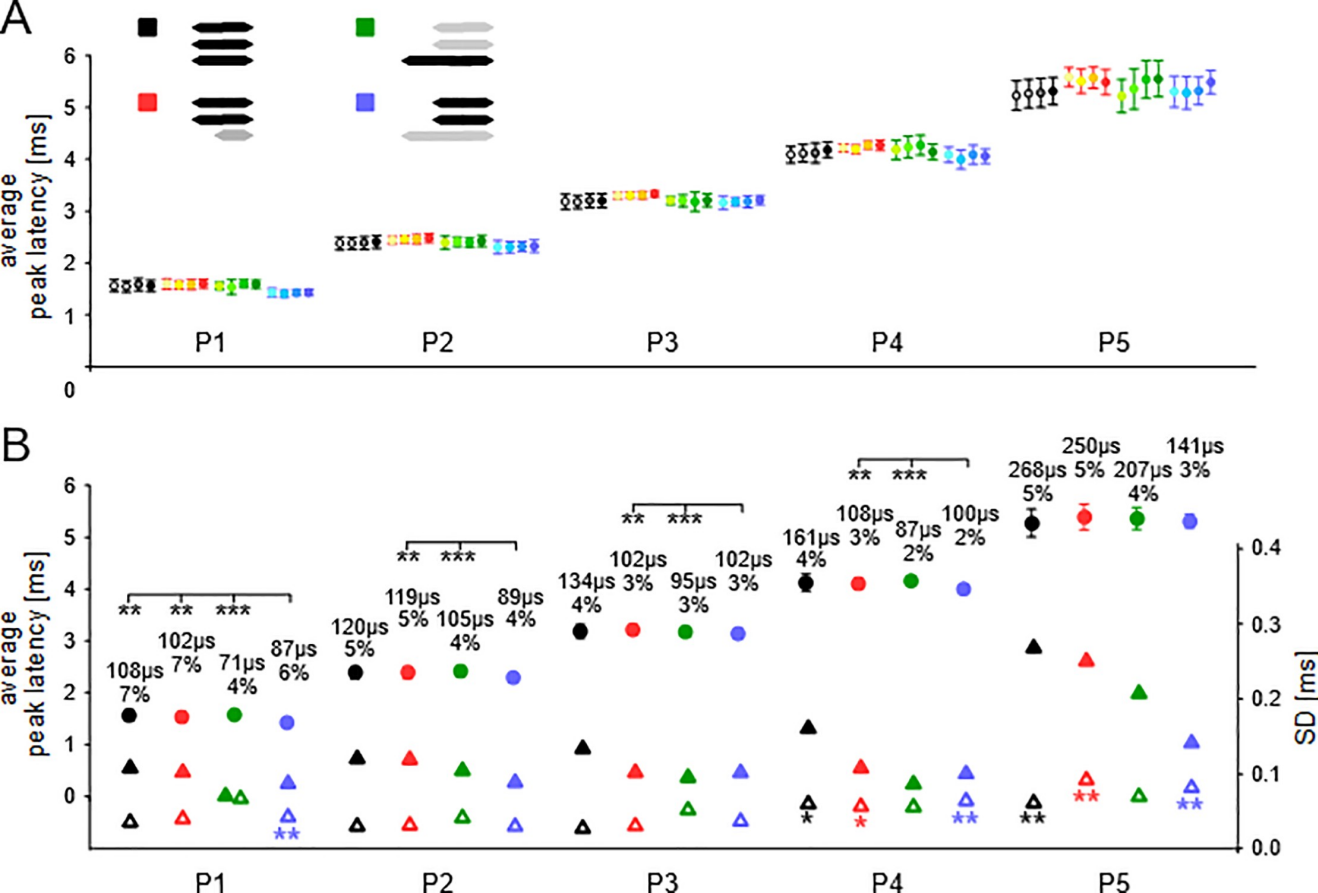
Experiment D, data evaluation. **(A)** Average latencies with SDs to the four sound bursts at the five wave peaks (P1-P5) when the three harmonics of the wriggling call models started simultaneously (black symbols), responses to the higher harmonics (7.6 + 11.4 kHz) with delayed onset of 3.8 kHz (orange-red), responses to 3.8 kHz preceding the onset of the higher harmonics (green), and responses to the higher harmonics when their onset was preceded by the onset of 3.8 kHz (blue). The shown average latencies include all cases of delayed onsets of 3.8 kHz (-10, -20, -30, -50 ms) and all cases of preceding onsets of 3.8 kHz (+10, +20, +30, +50 ms) relative to the higher harmonics. (**B)** Grand average latencies (explanation, see text) with SDs to the stimuli color-coded as in panel A (filled circles) at the five peaks (P1-P5). Asterisks indicate significant latency differences between responses to the blue stimulus and the other stimuli. The SDs of the means are also shown as values, relative values (%) and as filled triangles on the SD scale (right y-axis). The average SDs of the individual animals at the wave peaks are indicated by open triangles. The asterisks mark significantly larger values at P4 and P5 compared to those at P1, P2 and P3 and at P1 compared to P2 (see also Table 2).

Example ABR recordings to series of four bursts of wriggling call models with ISIs of 200 ms duration and non-simultaneous onsets of the harmonics are shown in the S4 Fig. The results of the quantitative data evaluation are shown in Fig 7. Latency means with SDs of the means, averaged across 8 animals, are shown in Fig 7A for P1-P5 separately for the 4 cases of responses to simultaneous onset of the three harmonics (black), responses to the higher harmonics (7.6 + 11.4 kHz) with delayed 3.8 kHz onset (orange-red), responses to 3.8 kHz preceding the onset of the higher harmonics (green), and responses to the higher harmonics when their onset was preceded by the 3.8 kHz onset (blue). The average latencies include all cases of delayed (-10, -20, -30, -50 ms) and preceding (+10, +20, +30, +50 ms) onsets of 3.8 kHz relative to the higher harmonics because there were no differences (ANOVA, p > 0.05) of average latencies between all these cases at any peak (see S5 Fig). Therefore at a given peak, latencies were averaged across delay/advance times separately for each of the four sound bursts for each animal. These average values from the 8 animals were then averaged to the average latencies plotted in Fig 7A with the respective SDs for each burst. Fig 7A shows clearly that, at a given peak, mean latencies and SDs of the means were very similar among the responses to the four sound bursts and were also rather similar with regard to the four different stimulus situations.

Since mean latencies in response to each of the four sound bursts did not differ in any of the four stimulus situations (ANOVA, p > 0.2), we averaged the average response latencies across delay/advance times and the four sound bursts for each animal, averaged the values of the animals and plotted the grand average latencies with the SDs of the means for all peaks and stimulus situations in Fig 7B (filled circles with SDs). The mean latencies increased through the sequential peaks from P1 to P5 resulting in a total latency increase of 3.729 ms (simultaneous onset of the three harmonics, black), 3.831 ms (higher harmonics 7.6 + 11.4 kHz with delayed onset of 3.8 kHz, red), 3.785 ms (3.8 kHz preceding the onset of the higher harmonics, green), and 3.884 ms (higher harmonics when their onset was preceded by the onset of 3.8 kHz, blue). The values of the absolute and relative SDs are noted above the means. As indicated in Fig 7B, there were statistically significant differences between the mean latencies from the four stimulus situations at P1, P2, P3, and P4 (ANOVA on ranks followed by U-test). The mean latencies of the responses to the two higher harmonics with preceding 3.8 kHz (blue) were shorter (p < 0.01) than the latencies to the two higher harmonics with delayed 3.8 kHz (red), shorter (p < 0.001) than the latencies to 3.8 kHz when the higher harmonics were delayed (green) and, only at P1, also shorter (p < 0.01) than the latency to the simultaneous onset of the three harmonics (black). For all four stimulus situations, the absolute SDs of the means were similar at P1-P4 but increased at P5 (filled triangles). The relative SDs of the means varied between 2% and 7%.

The mean SDs averaged from the individual SDs of the 8 animals are also shown in Fig 7B (open triangles). As in experiments A-C, individual SDs were generally smaller than the SDs of the means. The values of the average individual SDs (all peaks, all stimulus situations) ranged between 28 and 80 μs. The values at P4 and P5 were significantly larger than those at P1, P2, and P3 (ANOVA or ANOVA on ranks followed by t-test or U-test) for the stimulus situations of simultaneous onset of the harmonics (black), higher harmonics with delayed onset of 3.8 kHz (red), and higher harmonics with preceding onset of 3.8 kHz (blue). In the latter case, also average individual SDs at P1 were significantly larger than at P2. The respective levels of significance are indicated by the number of stars on the open triangles at P1, P4 and P5 in Fig 7B.

In summary, the main effect of non-simultaneous onsets of the harmonics of wriggling call models was the shortened latencies at P1-P4 of responses to the higher harmonics with preceding first harmonic (3.8 kHz) when compared with the responses to the other stimulus situations. Interestingly, the amount of the advanced 3.8 kHz onset in the tested range of 10–50 ms did not influence the amount of shortening the latencies of the responses to the delayed higher harmonics.

## Discussion

### Significance of ABR latency variability

Mouse ABR references from young, normal hearing mice using clicks, bursts of noise or tones of at least 50 dB SPL [32,33,35,36,40,42–44,60,61] indicate that our latency data (Table 1) and SDs of the means (see Figs 3B and 3C, 5, 7) were in the expected range of 1–2 ms latency for P1 increasing to 4.5–6.2 ms for P5 with SDs of the means generally increasing with ordinal numbers of peaks from 40–200 μs at P1 to more than 200 μs at P5. Standard deviations of ABR response latencies on the level of individual animals seem not to be available in the literature. SDs of the individual animals were mostly smaller than 100 μs and not generally increasing with increasing peak number (Table 2). SDs of individuals can be expected to be much smaller than SDs of population means since the latter reflect differences between the individuals concerning factors such as the physiological condition of the animals, placement and contact of electrodes, depth of anesthesia, and signal-to-noise ratio of the recording.

Remarkably, the SDs of the individuals expressing 30–90 μs latency jitter at the wave peaks (Table 2) reproduce the jitter in single neuron latency measurements from electrical stimulation of brain slices of the mouse. This jitter was 27 μs for excitatory postsynaptic potentials of CN octopus cells [62], 70 μs for first spikes of neurons in the medial nucleus of the trapezoid body [63], and about 100 μs for first spikes of rat IC cells (neurons with the least jitter) stimulated via a CN implant [64]. In addition, the decrease of the values of the SDs of the individuals from P1 to P2 (Table 2) may reflect the decrease of jitter of action potential timing of the neurons represented by P2 in the CN compared to the AN. Expressed by a synchronization index, neurons in the CN synchronized their action potentials 20–40% more precisely than AN fibers to the phase of low-frequency tones or to the amplitude modulation of sounds [65,66]. In correlation with this improvement of action potential synchronization, values of individual SDs at P2 were 30–40% smaller than the values at P1 (Table 2; SD individuals at 3,8 kHz, ‘C, D no adaptation’, ‘D enhanced’).

In conclusion, latency jitter of individual animals at the ABR peaks seems to reproduce the precision of response timing including adaptation (see below) of groups of neurons in the cochlea and auditory brain centers responsible for the generation the ABR waves. Thus, the evaluation of SDs of ABR latencies of individual subjects may open a wide new field for ABR studies by offering a simple method for detecting subtle changes in the precision of response timing at the neuronal level related to influences such as physiological condition, attention, behavioral and acoustic context, drugs, and genetic variation.

### Significance of ABR latency differences

Besides the expected latency differences of about 1 ms between the wave peaks (Table 1), which relate to synaptic levels in the ascending auditory pathway [28,29,32,34], we found an average latency difference of 0.342 ms related to P1 when generated by high (50 kHz) vs. low (3.8 kHz) tone stimulation (Table 1, Δ *D*-*A*). The frequency of 3.8 kHz is at the low end, 50 kHz at the high end of characteristic frequencies of AN fibers and IC neurons in NMRI mice [67,68] and other mouse strains [69–71]. Average first-spike latencies of AN fibers of very low and high characteristic frequencies differ by 1.03 ms (CBA/CaJ mouse [72] with citation of MC Liberman, personal communication) or 1.34 ms (NMRI mouse) [67]. Synaptic and neural processes in the cochlea take about 1 ms regardless of the characteristic frequency [73]. The front of the traveling wave induced by a tone takes about 0.3–0.4 ms to reach a place 6.8 mm from the cochlear base in direction towards the apex on the 20 mm long chinchilla basilar membrane [73–75]. The basilar membrane length of the NMRI mouse is 6.8 mm [76]. Therefore, the chinchilla data suggest that the ABR latency difference of 0.342 ms at P1 in the NMRI mouse may just reproduce the 0.34 ms latency difference between high- and low-frequency AN fibers [67] in this mouse strain after the 1 ms for synaptic and neural processes in the cochlea have been subtracted from the AN fibers latency difference. Thus, our data offer a reasonable value for the cochlear travel time in the mouse that has not yet been determined experimentally.

### Significance of ABR latency adaptation

Neural adaptation to series of sounds and recovery from adaptation have been investigated via several neuronal response parameters such as latencies, amplitudes, rates, and thresholds. We are not aware of data on adaptation of latency jitter as expressed by SDs of latencies of individual animals (Fig 6C). In general, adaptation effects such as those found in experiment C (Figs 5 and 6) increase with decreasing ISI duration and increasing level of processing in the auditory system [21,26,38,77–82]. As we show in Figs 5A and 6A, the most prominent adaptation effect is seen in the response to the second sound in a series of identical sounds [83–86]. In the auditory brainstem, the amount of adaptation as a function of the ISI duration follows exponential decreases or linear decreases on a logarithmic scale in the range of up to about 200 ms ISIs [85,87–89] as shown in Fig 6A and 6B and Table 3 for all peaks of the mouse ABR. We found longer latencies from adapted compared with non-adapted responses in experiments C and D for P2-P5, statistically significant for P2-P4 (Table 1). One may argue that, at each center of the auditory pathway represented by the ABR peaks, adaptation could add similar amounts of latency to the sound onset responses conveyed centripetally. Interestingly, however, this is not the case. The centers of the auditory pathway generating the ABR wave peaks (P1-P5) did not contribute equally to the total adaptation effect of 0.254 ms latency increase measured over the peaks in the adapted versus the non-adapted cases (see Table 1, Δ value at P5-1 for ‘C, D no adaptation’ vs. ‘C adaptation’). The transition from P1 (cochlea) to P2 (groups of neurons in the CN) contributed about 2/3 (0.162 ms, see Δ value at P2-1 in Table 1) to the total adaptation-related latency increase over the peaks, while other transitions contributed less or nothing. That is, ABR latency differences express adaptation effects specific for the transition from one to another processing center responsible for the given ABR peaks.

One initial question of this study concerned possible relationships between changes of ABR latencies/latency precision and boundaries in the time domain near 20–30 ms, 100 ms, and 400 ms known from sound perception in humans, mice, and other animals [3]. The results of our study showed:

1. The perceptual boundary near 20–30 ms depending on the *duration of sounds or sound components* [11,14,49] (experiments A, D) had no counterpart in our results.
2. The perceptual boundary near 20–30 ms depending on the *duration of inter-sound intervals* [1–4,8–10] was reflected in the significant adaptation of latencies and the amount of latency jitter at P2-P5 for ISIs < ~30 ms (experiment C, Figs 5A, 6A and 6C, Table 3). Our experiment B showed that relatively soft noise bursts (60 dB total SPL or 43.6 dB in the critical band of 23 kHz around 50 kHz [90]) did not significantly adapt onset responses to subsequent 50 kHz tones at any of the ISIs (2–100 ms) tested. Transferred to coding of human speech sounds, the noise bursts due to consonants such as b, d, g, k, p, t, are not expected to have adaptation effects in the brainstem on the coding of vowels vocalized at the end of VOTs. Experiments with chinchillas support this hypothesis. Chinchilla AN fibers [91,92] and part of the neurons in the IC [93] reliably encoded with their onset-response latencies synthetized vowels after VOTs of 20 ms and longer especially when the neurons responded preferentially to the lowest formant frequency of the vowel.
3. The perceptual boundary near 100 ms depending on the *duration of inter-sound intervals* [15,16] was reflected in the beginning of significant adaptation of latencies and the increase of latency jitter at P5 for ISIs < ~100 ms (experiment C, Figs 5A, 6A and 6C). Thus, we can state that optimal perception of wriggling call series in one acoustic stream with ISIs > 100 ms happens when the sound onset responses at P5, most probably representing IC-responses in the mouse [32–34], are largely free from adaptation and able to follow with full temporal precision the sound onsets. Likewise in human perception, series of two tones with frequencies within one critical band are integrated in one perceptual stream, if the ISIs are longer than about 100 ms [15]. In this case, adaptation and loss of temporal precision of sound onset coding in critical frequency bands related to the IC, which was found to be the first level in the auditory pathway with neural correlates of critical band properties [94,95], may have prevented the two frequencies be integrated in one perceptual stream when ISIs were shorter than about 100 ms.
4. The 400 ms boundary in perception did not occur in our ABR latency data. We did not find adaptation effects for ISIs longer than about 200 ms in any of the auditory centers examined (Figs 5A, 6A and 6C). In addition, in the auditory cortex enhanced responding is seen to a sound which is preceded by another sound with ISIs of 400 ms or less [96–98]. If such an enhancement were already established in brainstem centers, we might have recorded ABR responses with increased amplitude and shortened latency to the onset of the second, third, and forth compared to the first wriggling call models in series with 100 ms or 200 ms ISIs in experiment C. This was, however, not the case. Therefore, our guess is that the 400 ms ISI boundary is established in higher auditory centers above the auditory midbrain.

In conclusion, adaptation of ABR latencies means a loss in the precision of coding sound onsets. The amounts of latency adaptation and loss of coding precision increase nonlinearly with shortening of intervals between sounds in a series and nonlinearly with increasing processing levels in the auditory pathways. At the levels of the auditory brainstem and of the auditory midbrain, latency adaptation with loss of coding precision for sound onsets reflects the ISI boundaries in the perception of sound streams near 20–30 ms and 100 ms, respectively.

### Significance of ABR latency enhancement

The 10–50 ms earlier onset of 3.8 kHz relative to the higher harmonics (7.6 + 11.4 kHz) of the wriggling call models in experiment D had no adaptation effects on the responses to 7.6 + 11.4 kHz. To the contrary, the mean onset response latencies decreased by about 0.1 ms at each peak compared to those of the other stimulus cases shown in Fig 7 (blue data compared to green, red and black data). This significantly enhanced state of the response to the higher harmonics is also shown in Table 1, where response latencies without adaptation (‘C, D no adaptation’) were compared with those of the enhanced responses (‘D enhanced’). Our approach to explain these data of experiment D has to consider processes starting in the cochlea because the shortening of response latencies started with P1 and was carried on to the next peaks.

As mentioned above, adaptation effects on the perception of auditory streams [15] seem to concern only frequencies within one given critical band. Since the higher harmonics of wriggling calls were outside the critical band determined by 3.8 kHz [54,90] adaptation of responses to the higher harmonics by 3.8 kHz was not expected. The enhancement of responses to the higher harmonics may have been caused due to the excitation patterns of the cochlea [99,100] because the 3.8 kHz tone of 60 dB SPL stimulated the cochlear locations where the responses to 7.6 + 11.4 kHz were generated according to the tonotopic gradient [71,101]. Masking experiments with auditory nerve fibers have shown that such stimulation by low-frequency and low-level tones or noise adds excitation to the tonotopically proper stimulus of higher frequencies leading to increased response rates [67,102,103]. Considering the general relationship between increased response rates and shortening of onset response latencies at moderate sound intensities for neurons in the AN and auditory brainstem [64,104–107], we have the explanation for the enhanced response to the higher harmonics at P1 when 3.8 kHz was preceding their onset by any of the tested advanced onset times. This enhancement was then continued as significant latency decrease up to P4 and faded away at P5, where the latency variation was increased (Fig 7). With regard to wriggling call perception, the presence of 3.8 kHz in combination with one or both higher harmonics in the sound stimulus significantly enhanced call perception compared with stimuli containing only higher harmonics [54]. Similarly, in human vowel perception low-frequency formants below the best hearing range of about 2–5 kHz [108] have been shown to be most important [109,110].

In summary, the latency results from our experiments C and D indicate adaptation effects when the frequencies in a stream of sounds of short ISIs were within the same critical band, and enhancement effects of low-frequency on high-frequency sound components when the frequencies concerned different critical bands. This enhancement may be important for perception of multi-harmonic communication sounds such as vowels in human speech.

## Supporting information

S1 Fig Experiment A, average peak latencies as function of ultrasound duration. Latencies with standard deviations averaged from the responses to the four 50 kHz bursts of the 6 experimental animals are plotted at the wave peaks P1–P5 separately for all tested durations of the 50 kHz ultrasound bursts. Significant differences between latencies at the sound durations did not occur at any peak.
(DOCX)

S2 Fig Experiment B, example ABR recording to noise-ultrasound pairs with ISIs of 100, 20, 8, and 2 ms duration. The ISIs are indicated by grey vertical bars. The onset of the ultrasound after the ISIs is shown by an arrow followed by a hatched bar marking the first millisecond on the ultrasound scale. Absolute amplitudes of ABRs to noise were about twice as large as those to ultrasounds and relative amplitudes of the 5 waves, i.e. waveforms, differed between responses to noise and ultrasounds.
(DOCX)

S3 Fig Experiment C, example ABR recording to series of four wriggling call models. The ISIs between the sound bursts were either 200 ms or 20 ms (grey vertical bars). Wave peaks P1-P5 are indicated. In the responses to burst 1 (200 ms ISI) and bursts 3 and 4 (20 ms ISI), P5 could not be addressed and no latency measurements were taken.
(DOCX)

S4 Fig Experiment D, example ABR recording to series of four sound bursts mimicking wriggling call models with non-simultaneous onsets of the harmonics. (**A)** Responses to the 7.6 + 11.4 kHz harmonics when the onset of 3.8 kHz was delayed by 50 ms. (**B)** Responses to 3.8 kHz when the onset of 3.8 kHz preceded the onset of the higher harmonics by 50 ms. (**C)** Responses to the 7.6 + 11.4 kHz harmonics when 3.8 kHz started 50 ms in advance. The amplitudes of the ABR to the 3.8 kHz harmonic, when it was delayed with regard to the higher harmonics (panel A), were rather small so that latencies could not reliably be measured.
(DOCX)

S5 Fig Experiment D, average peak latencies for the responses to 7.6+11.4 kHz (circles) or 3.8 kHz (triangles) at the indicated delayed or preceding onset times of 3.8 kHz relative to the onsets of 7.6+11.4 kHz. Latencies with standard deviations averaged from the responses to the four sound bursts of the 8 experimental animals are plotted at the wave peaks P1–P5 separately for all tested delayed and preceding onset times of 3.8 kHz. Significant differences between latencies of the responses to 7.6+11.4 kHz did not occur at any peak when the onset of 3.8 kHz was delayed or preceding for the indicated times. Also, significant differences between latencies of the responses to 3.8 kHz did not occur at any peak when the onset of 3.8 kHz was preceding for the indicated times.
(DOCX)
