# Supplementary material for: Adaptation and spectral enhancement at auditory temporal perceptual boundaries - Measurements via temporal precision of auditory brainstem responses
Source: PLoS One. 2018 Dec 20;13(12):e0208935. doi: 10.1371/journal.pone.0208935 (PMC6301773; doi:10.1371/journal.pone.0208935)
Supplement: S1 Fig — Latencies with standard deviations averaged from the responses to the four 50 kHz bursts of the 6 experimental animals are plotted at the wave peaks P1–P5 separately for all tested durations of the 50 kHz ultrasound bursts. Significant differences between latencies at the sound durations did not occur at any peak. (DOCX) [file pone.0208935.s001.docx]

**
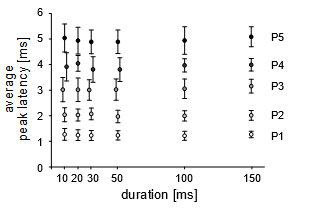
**

**Supplementary Figure 1. Experiment A, average peak latencies as function of ultrasound duration.** Latencies with standard deviations averaged from the responses to the four 50 kHz bursts of the 6 experimental animals are plotted at the wave peaks P1–P5 separately for all tested durations of the 50 kHz ultrasound bursts. Significant differences between latencies at the sound durations did not occur at any peak.
