# Supplementary material for: Adaptation and spectral enhancement at auditory temporal perceptual boundaries - Measurements via temporal precision of auditory brainstem responses
Source: PLoS One. 2018 Dec 20;13(12):e0208935. doi: 10.1371/journal.pone.0208935 (PMC6301773; doi:10.1371/journal.pone.0208935)
Supplement: S2 Fig — The ISIs are indicated by grey vertical bars. The onset of the ultrasound after the ISIs is shown by an arrow followed by a hatched bar marking the first millisecond on the ultrasound scale. Absolute amplitudes of ABRs to noise were about twice as large as those to ultrasounds and relative amplitudes of the 5 waves, i.e. waveforms, differed between responses to noise and ultrasounds. (DOCX) [file pone.0208935.s002.docx]

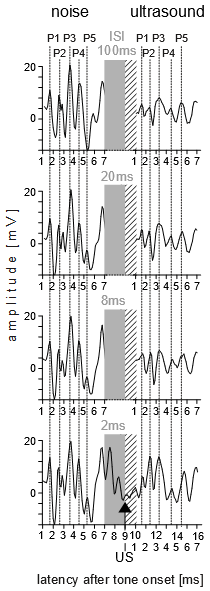


**Supplementary Figure 2. Experiment B, example ABR recordings to noise-ultrasound pairs**. The ISIs are indicated by grey vertical bars. The onset of the ultrasound after the ISIs is shown by an arrow followed by a hatched bar marking the first millisecond on the ultrasound scale. Absolute amplitudes of ABRs to noise were about twice as large as those to ultrasounds and relative amplitudes of the 5 waves, i.e. waveforms, differed between responses to noise and ultrasounds.
