# Supplementary material for: Adaptation and spectral enhancement at auditory temporal perceptual boundaries - Measurements via temporal precision of auditory brainstem responses
Source: PLoS One. 2018 Dec 20;13(12):e0208935. doi: 10.1371/journal.pone.0208935 (PMC6301773; doi:10.1371/journal.pone.0208935)
Supplement: S3 Fig — The ISIs between the sound bursts were either 200 ms or 20 ms (grey vertical bars). Wave peaks P1-P5 are indicated. In the responses to burst 1 (200 ms ISI) and bursts 3 and 4 (20 ms ISI), P5 could not be addressed and no latency measurements were taken. (DOCX) [file pone.0208935.s003.docx]

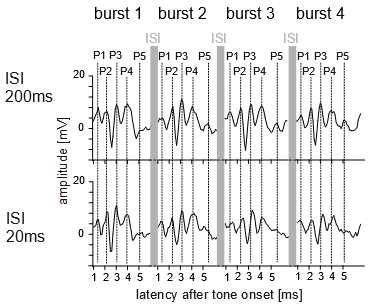


**Supplementary Figure 3. Experiment C, example ABR recording to series of four wriggling call models**. The ISIs between the sound bursts were either 200 ms or 20 ms (grey vertical bars). Wave peaks P1-P5 are indicated. In the responses to burst 1 (200 ms ISI) and bursts 3 and 4 (20 ms ISI), P5 could not be addressed and no latency measurements were taken.
