# Supplementary material for: Adaptation and spectral enhancement at auditory temporal perceptual boundaries - Measurements via temporal precision of auditory brainstem responses
Source: PLoS One. 2018 Dec 20;13(12):e0208935. doi: 10.1371/journal.pone.0208935 (PMC6301773; doi:10.1371/journal.pone.0208935)
Supplement: S4 Fig — (A) Responses to the 7.6 + 11.4 kHz harmonics when the onset of 3.8 kHz was delayed by 50 ms. (B) Responses to 3.8 kHz when the onset of 3.8 kHz preceded the onset of the higher harmonics by 50 ms. (C) Responses to the 7.6 + 11.4 kHz harmonics when 3.8 kHz started 50 ms in advance. The amplitudes of the ABR to the 3.8 kHz harmonic, when it was delayed with regard to the higher harmonics (panel A), were rather small so that latencies could not reliably be measured. (DOCX) [file pone.0208935.s004.docx]

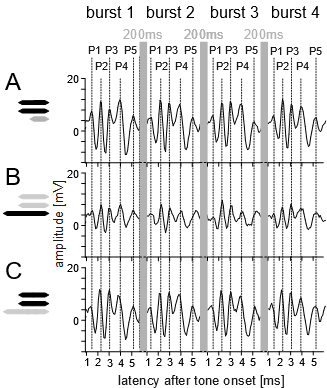


**Supplementary Figure 4.** **Experiment D, example ABR recording to series of four sound bursts**. (**A)** Responses to the 7.6 + 11.4 kHz harmonics when the onset of 3.8 kHz was delayed by 50 ms. (**B)** Responses to 3.8 kHz when the onset of 3.8 kHz preceded the onset of the higher harmonics by 50 ms. (**C)** Responses to the 7.6 + 11.4 kHz harmonics when 3.8 kHz started 50 ms in advance. The amplitudes of the ABR to the 3.8 kHz harmonic, when it was delayed with regard to the higher harmonics (panel A), were rather small so that latencies could not reliably be measured.
