# Supplementary material for: Adaptation and spectral enhancement at auditory temporal perceptual boundaries - Measurements via temporal precision of auditory brainstem responses
Source: PLoS One. 2018 Dec 20;13(12):e0208935. doi: 10.1371/journal.pone.0208935 (PMC6301773; doi:10.1371/journal.pone.0208935)
Supplement: S5 Fig — Latencies with standard deviations averaged from the responses to the four sound bursts of the 8 experimental animals are plotted at the wave peaks P1–P5 separately for all tested delayed and preceding onset times of 3.8 kHz. Significant differences between latencies of the responses to 7.6+11.4 kHz did not occur at any peak when the onset of 3.8 kHz was delayed or preceding for the indicated times. Also, significant differences between latencies of the responses to 3.8 kHz did not occur at any peak when the onset of 3.8 kHz was preceding for the indicated times. (DOCX) [file pone.0208935.s005.docx]

**
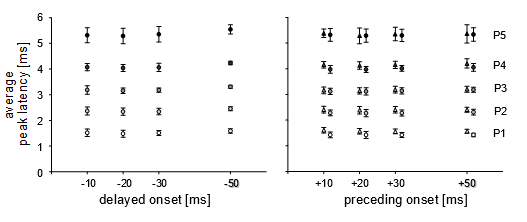
**

**Supplementary Figure 5. Experiment D, average peak latencies for the responses to 7.6+11.4 kHz (circles) or 3.8 kHz (triangles) at the indicated delayed or preceding onset times of 3.8 kHz relative to the onsets of 7.6+11.4 kHz.** Latencies with standard deviations averaged from the responses to the four sound bursts of the 8 experimental animals are plotted at the wave peaks P1–P5 separately for all tested delayed and preceding onset times of 3.8 kHz. Significant differences between latencies of the responses to 7.6+11.4 kHz did not occur at any peak when the onset of 3.8 kHz was delayed or preceding for the indicated times. Also, significant differences between latencies of the responses to 3.8 kHz did not occur at any peak when the onset of 3.8 kHz was preceding for the indicated times.
